# The Automated Systematic Search Deduplicator (ASySD): a rapid, open-source, interoperable tool to remove duplicate citations in biomedical systematic reviews

**DOI:** 10.1186/s12915-023-01686-z

**Published:** 2023-09-07

**Authors:** Kaitlyn Hair, Zsanett Bahor, Malcolm Macleod, Jing Liao, Emily S. Sena

**Affiliations:** Centre for Clinical Brain Sciences, University of Edinburgh, Edinburgh, UK

**Keywords:** Automation tools, Living systematic reviews, Deduplication, Systematic search, Bibliographic database, Citation manager, EndNote, Systematic reviews

## Abstract

**Background:**

Researchers performing high-quality systematic reviews search across multiple databases to identify relevant evidence. However, the same publication is often retrieved from several databases. Identifying and removing such duplicates (“deduplication”) can be extremely time-consuming, but failure to remove these citations can lead to the wrongful inclusion of duplicate data. Many existing tools are not sensitive enough, lack interoperability with other tools, are not freely accessible, or are difficult to use without programming knowledge. Here, we report the performance of our Automated Systematic Search Deduplicator (ASySD), a novel tool to perform automated deduplication of systematic searches for biomedical reviews.

**Methods:**

We evaluated ASySD’s performance on 5 unseen biomedical systematic search datasets of various sizes (1845–79,880 citations). We compared the performance of ASySD with EndNote’s automated deduplication option and with the Systematic Review Assistant Deduplication Module (SRA-DM).

**Results:**

ASySD identified more duplicates than either SRA-DM or EndNote, with a sensitivity in different datasets of 0.95 to 0.99. The false-positive rate was comparable to human performance, with a specificity of > 0.99. The tool took less than 1 h to identify and remove duplicates within each dataset.

**Conclusions:**

For duplicate removal in biomedical systematic reviews, ASySD is a highly sensitive, reliable, and time-saving tool. It is open source and freely available online as both an R package and a user-friendly web application.

**Supplementary Information:**

The online version contains supplementary material available at 10.1186/s12915-023-01686-z.

## Background

### The need for effective and efficient deduplication

Researchers performing high-quality systematic reviews typically search across multiple biomedical databases to collect as many relevant citations as possible [1]. This process can introduce a substantial number of duplicate citations [2]. For example, overlap between EMBASE and PubMed is estimated to be as much as 79% [3].

Effective duplicate removal is an essential, if underappreciated, part of the data identification process of systematic reviews [4, 5]. If duplicate citations are not removed effectively, reviewers can waste time screening the same citations for inclusion, and run the risk of accidentally including the same paper more than once in their meta-analyses, potentially leading to inaccurate conclusions [6]. Anecdotally, we recently came across remaining duplicate citations during the analysis stage of a large scale and highly collaborative systematic review [7]. In such cases, there is a realistic probability of duplicate citations progressing through screening and data extraction undetected, as different reviewers may handle each citation. The incorrect removal of citations that are not duplicates (false positives) is equally problematic. Through the erroneous removal of relevant citations, false positives can introduce biases and reduce the reproducibility of systematic reviews [8–10].

### Current deduplication approaches

To address the need for duplicate removal, researchers often use citation management software or stand-alone applications. An overview of commonly used approaches is shown in Table 1.

**Table 1:** Current deduplication tools and approaches for systematic reviews

Tool	Description	Resource required	Accessibility	Performance
EndNote [11]	Citation manager	**Medium** (requires manual effort to improve sensitivity)	**Low** (requires a paid subscription)	**Low**—medium (user-dependent) [4, 5, 8, 12]
SRA-DM [12]	Web/desktop application	**Low**	**High**	**High**
Revtools [13]	R package with functions: find_duplicates()	**Medium** (users set threshold for deduplication)	**Medium** (R knowledge required)	Unknown
bibliometrix [14]	R package with functions: duplicatedMatching() mergeDbSources()	**Medium** (users set threshold for deduplication; ability to merge Web of Science and Scopus citations)	**Medium** (R knowledge required)	Unknown
synthesisr [13]	R package with functions: deduplicate()	**Medium** (users set threshold for for deduplication)	**Medium** (R knowledge required)	Unknown
Metta [15]	Cross database search engine	**Low**	**Medium** (not openly accessible)	**High** [9]
Zotero [16]	Citation manager	**Medium** (manual merging required)	**High**	**Low** [10]
Covidence	Systematic review platform	**Low**	**Low** (requires paid subscription)	**High** [10]
Mendeley [17]	Citation manager	**Medium** (manual merging required)	**High**	**High** [8, 10]
Hand-searching	Manual	**High**	**Low**	**High** [4]

Several citation managers offer automated matching algorithms which then allow users to manually decide on potential duplicate matches within their search dataset. Few solutions offer a “one-click” duplicate removal option. EndNote is one of the most established citation managers [18] and can automatically detect citations matching on “Author”, “Year” and “Title” and remove them without additional manual checking. However, evidence suggests automated deduplication in EndNote is not sufficient and fails to identify as many duplicates as other methods [2, 8, 12]. Several other tools for removal of duplicates have emerged in recent years, either as stand-alone tools or as part of alternative workflows (which may bypass the need for traditional citation managers). The Systematic Review Assistant (recently renamed and upgraded to Systematic Review Accelerator, SRA2) is a suite of free, open-source systematic review tools developed by researchers at Bond University. Their “Deduplication Module” (SRA-DM) has a user-friendly interface in which users can upload a search file in various formats and perform automated duplicate removal in a few clicks. Previously, SRA-DM was shown to identify substantially more duplicates than EndNote [12]. Where identifying the greatest number of citations is paramount, manual deduplication by hand-searching (often in combination with citation management software) can be a highly effective approach [2] but becomes impractical with particularly large search datasets.

When considering the performance of different tools to identify duplicates from systematic search datasets, we must also take into account what type of citation data the tool was designed to deduplicate. The type and extent of duplicate publications may differ—an author may publish a higher number of similar papers in a short space of time, or there may be less bibliometric information available for studies published in lesser known (and less frequently indexed) journals. Most currently available tools have been designed for clinical systematic reviews and may peform differently in other domains.

Finally, many deduplication tools are proprietary software which restricts their accessibility, prevents intereroperability, and limits transparency about how their underlying duplicate detection process works. Increasingly, freely available open-source citation managers such as Zotero and Mendeley have gained popularity.

### Addressing unmet deduplication needs for preclincial systematic reviews

In research-intensive fields with a rapid rate of publication, the number of potentially relevant citations identified in a systematic search can be extremely large. For example, we frequently retrieve tens of thousands of potentially relevant citations for preclinical systematic reviews of experiments modelling neurological disease in animal models [19]. Previous evaluations of duplicate removal tools have used relatively small (< 5000 citations) systematic search datasets primarily representing clinical research citations [2, 8, 12]. Deduplication tools should therefore be evaluated on comparatively large datasets to determine the magnitude of gains and losses on that scale (e.g. how many duplicate citations a tool is likely to remove correctly). There is also a clear need for deduplication tools which have been validated in non-clinical search datasets (e.g. for preclinical systematic reviews). Due to the sheer volume of publications identified in some reviews, automated approaches with little manual resource required are warranted and preferable.

Increasingly, meta-researchers are aspiring to provide automated or “living” systematic reviews [20], producing real-time summaries of a research domain including the most recent research findings. To enable such summaries, we need automation tools at each stage of the review process that are reliable, interoperable, and require minimal manual intervention. Depending on the project goals, it may also be useful to have some control over the tool’s duplicate removal logic, e.g. to configure the tool to retain an older version of a citation when a new, matching citation is identified in an updated search. This approach could also be used in more conventional systematic review updates, often occurring after many years [21] and often involving significant overlap between systematic search dates to prevent missing relevant studies. Alternatively, researchers may wish to preferentially retain the newer citation, which may be more complete and may contain more accurate meta-data. Many citation mangers support the selection of which citation record to preserve, yet this is often a manual process for each duplicate group or pair.

To address some of these unmet needs required for deduplication for preclinical systematic reviews, we developed the Automated Systematic Search Deduplicator (ASySD). ASySD is an R–based tool that can also be accessed via a web application. To use ASySD, researchers do not need to learn R or have any knowledge of match parameters. In a few clicks, users can download a customisable unique citation dataset, without having to manually inspect and merge groups of duplicate citations. We aimed to critically evaluate the performance of ASySD in comparison with two comparatively user-friendly, low-effort automated tools which provide automated solutions to deduplication—EndNote’s automated duplicate removal functionality and Bond University’s SRA-DM.

## Methods

Prior to evaluating the performance of ASySD, we registered a protocol describing our methods on the Open Science Framework [22].

### Definition of “Duplicate citations”

We define duplicates as the presence of two or more citations representing the same bibliographic publication within an aggregated systematic review search result, even where those citations differ subtly in recorded details such as author(s), title, journal pagination, issue number, or volume. If the same study is published in two separate journals, we do not consider this a duplicate citation for these purposes. Similarly, sets of conference abstracts, preprints, and journal articles which describe the same research are not classed as duplicate citations. The SRA-DM tool’s definition of duplicates is consistent with ours. Importantly EndNote X9’s default configuration defines duplicates as citations which match on specific fields (title, author, year), which may impact on performance. For example, preprints and their subsequent journal articles often match on these fields, as do articles published across multiple journals.

### Tool development and functionality

We developed ASySD in the R programming language. To improve the chance of detecting duplicate citations, we process the data to undergo several cleaning and formatting steps as part of the tool. These includes renaming missing or anonymous Authors as “Unknown”, harmonising differences in DOI format, removing punctuation, and making all citation information upper case.

Using the RecordLinkage R package [23, 24], we applied blocking criteria (fields which must be a 100% match) to identify possible duplicate pairs. These criteria were largely based on guidance to systematically identify all possible duplicates using EndNote’s manual 100% match filters [5]. Blocking criteria (see Table 2) were applied in four separate rounds because of the extensive memory requirements needed to perform these operations on large datasets in R; however, matches identified within any of the rounds were considered a possible duplicate pair.

**Table 2:** Blocking criteria specified for ASySD to identify potential duplicate citations

Order	Blocking criteria (100% match on specified fields)
Round 1	(**Title** AND **Pages**) OR
	(**Title** AND **Author**) OR
	(**Title** AND **Abstract**) OR
	**DOI**
Round 2	(**Author** AND **Year** AND **Pages**) OR
	(**Journal** AND **Volume** AND **Pages**) OR
	(**ISBN** AND **Volume** AND **Pages**) OR
	(**Title** AND **ISBN**)
Round 3	(**Year** AND **Pages** AND **Volume**) OR
	(**Year** AND **Issue** AND **Volume**) OR
	(**Year** AND **Pages** AND **Issue**)
Round 4	(**Author** AND **Year**) OR
	(**Title** AND **Year**) OR
	(**Title** AND **Volume**) OR
	(**Title** AND **Journal**)

Most pairs identified with blocking criteria are not true duplicates, and further comparisons are needed to ascertain duplicate status. To compare the overall similarity of a matching pair, we also calculate Jaro-Winkler string comparisons across all relevant fields (Title, Author, Year, Journal, ISBN, Abstract, DOI, Issue, Pages, and Volume) using the RecordLinkage package. Using a heuristic approach, we developed and applied additional match filters based on string comparison match strength (a numerical value between 0 and 1) to optimise performance and prevent the deletion of citations, which were not duplicates. During development, we used three existing CAMARADES systematic review search results with labelled duplicates (Neuropathic Pain [7], Antioxidants [25], and Epilepsy [26]) to iteratively validate and adjust the match filters to improve the performance of the tool.

Once ASySD identifies all matching citations, one citation is removed from each pair. First, citations which do not contain abstracts are preferentially removed. Where a newer version of a citation exists (e.g. e-publication date versus publication date), we will preferentially retain the most up-to-date version. If neither of these rules apply (e.g. both citations contain abstract text, and have the same year of publication), then the second listed citation in each pair is removed. Where there are more than two duplicates, the code logic ensures that only one is kept from within each duplicate set. There is also an option for users to determine which citations should be preferentially retained in the dataset using a “Label” field. When a matching citation is identified, the citations with a user-specified label (e.g. citations from an older search or citations from a certain database) will be retained over other citations.

Citation pairs which fall just short of the match filters—for example, citations pairs with matching DOIs or highly similar titles that have lower than expected similarity on journal or author—are retained for manual deduplication. As part of this functionality, users can manually review these matches and select which (if any) citation of the two they would like to remove from the search.

The underlying code for ASySD is open-source and available on GitHub, where it is also available to download as an R package [27]. To ensure accessibility, we have also created a user-friendly web application built using R Shiny (https://camarades.shinyapps.io/ASySD/). Users can upload a file with search returns (e.g. EndNote XML, RIS, BibTex), click a button to run the deduplication procedure, complete any additional manual deduplication within the application (if required), and download the results for import into various citation managers. There is also an option to download a file with the all of the original citations, with a group of two or more duplicates flagged with the same identifier for manual review. The code underlying the Shiny web application is also available on GitHub [27].

### Gold-standard systematic search datasets

We assessed the performance of automated deduplication tools on five test datasets of varying sizes from systematic review searches (Table 3). For each dataset, duplicate citations had been removed in EndNote using a combination of automated deduplication functions, changing field parameters to identify all citations which match on certain field, e.g. “Title”, and manual checking. Citations which had been removed by the human reviewer were reinstated and labelled as duplicates. We obtained three systematic search datasets from external sources, described below. We also used two datasets curated as part of ongoing in-house projects, a systematic review of systematic reviews of animal models of human disease (SRSR), and a systematic review of animal models of depression. Importantly, none of these datasets had been used in the development of the tool. To assess the time taken to perform “gold-standard” deduplication, we measured the time taken to deduplicate the SRSR dataset. To identify duplicates, we imported the systematic search into EndNote and followed recommended guidance [5] to systematically identify all duplicate citations in the dataset using a range of different matching field parameters, e.g. matching on “Author” and “Year”.

**Table 3:** Gold standard systematic search datasets

Dataset description	Databases searched	Citations obtained	Duplicates removed	Citations remaining
Diabetes dataset: Antidiabetics in animal models of atherosclerosis (SYRCLE, Radboud University) [28]	PubMed, EMBASE	1845	896	949
Neuroimaging dataset: Epigenetic neuroimaging (MRC Centre for Reproductive Health, University of Edinburgh) [29] (*Preclinical (*in vivo*) and clinical data included in review)*	SCOPUS, EMBASE, Medline, Web of Science,	3438	1280	2158
Cardiac dataset: Efficacy of cardiac ischemic preconditioning in animal models (SYRCLE, Radboud University) [30]	PubMed, EMBASE	8948	3153	5795
Depression dataset: Preclinical animal models of Depression (CAMARADES, University of Edinburgh) [31]	PubMed, EMBASE, Web of Science,	79,880	9418	70,462
Systematic review of systematic reviews (SRSR) dataset: Systematic review of preclinical systematic reviews dataset (CAMARADES, University of Edinburgh) [32]	PubMed, EMBASE, Web of Science,	53,001	16,778	36,223

### Methods for performance evaluation in testing datasets

To obtain the most up-to-date citation information and ensure all systematic searches for validation have a similar depth of information, we used the “Find Reference Updates” feature in EndNote X9 to retrieve additional information (e.g. DOIs, page numbers, issue numbers, journal volumes). Citations which had been removed by the human reviewers were labelled in the EndNote file.

We compared the performance of the ASySD tool (automated, with no manual input, deduplication mode only), EndNote X9 automatic deduplication, and SRA-DM offline application (Windows version of the front-end user interface [33]) on the five gold-standard search datasets. To assess auto-deduplication performance using EndNote X9, we auto-deduplicated citations based on “Author”, “Year” and “Title” matching criteria and using the “Ignore spacing and punctuation” feature. In SRA-DM, we uploaded XML files of our datasets to the offline version of the tool (as the server has limited capacity for high volume datasets) and chose the automated deduplication option to remove all suspected duplicates. In the ASySD tool, we uploaded citations as an XML file to a local version of the web application in RStudio Server version 1.4.1106 (R version 3.6.3) and ran automated deduplication. Because of memory limitations on the shinyapps.io server, for search results containing over 50,000 citations, we ran the R Shiny application locally in R Studio. We configured the ASySD application to preferentially remove citations which had also been labelled as duplicates by the human reviewer. Importantly, this process does not affect the number of duplicate pairs identified by the tool—only the choice of which citation from each pair/ group of citations is removed. Rather than removing citations with less information (e.g. no abstract), the tool preferentially selected the one the human reviewer had removed. This allowed us to compare the performance of the human reviewer vs ASySD more easily. When evaluating the tool, we found that under certain circumstances, this setting results in slightly fewer duplicates where there are many duplicates of one citation, but not all of these duplicates are paired with each other (e.g. when citation A = citation B, citation B = citation C, citation C = citation D, but citation D does not match with citation A or B). The impact of this is very minor and unlikely to meaningfully alter performance metrics. This discrepancy was identified only in the two larger datasets, with 13/79,880 (Depression dataset) and 13/53,001 (Systematic review of systematic reviews dataset) additional citations retained using the human labelled citation preferentially versus the default deduplication settings (Additional File 1).

Once duplicates were removed using each of the other tools, a “Duplicate ID” was generated for matching sets of duplicates identified by ASySD. This was possible because ASySD allows users to download the Record IDs of matching citation pairs. For each Duplicate ID, there should therefore be one single citation labelled as “KEEP” and the remainder (one or more duplicate citations) labelled as “REMOVE”. We carried out extensive manual checking in MS Excel to interrogate duplicate citations identified by some approaches but missed by others, to ensure that they were indeed duplicates. We manually searched to identify additional studies and corrected the Duplicate ID as appropriate. All data (including the original de-duplicated search datasets, results from each deduplication tool, final manually checked datasets with duplicate IDs, and the R code used to assess performance) are available to view on the Open Science Framework [34]. Once each search file had been corrected, we analysed this final dataset in R to calculate performance.

Since putting the first version of this work online as a preprint and making all underlying data available, an external researcher (GL) contacted us to inform us of missing duplicates and minor errors in our dataset. Following this, we re-checked each of the datasets and incorporated the necessary changes.

We report the performance of each tool by calculating:

- *Number of true positives* (citations which are duplicates which are correctly removed from the dataset);
- *Number of false positives* (citations which are not duplicates which are wrongly removed from the dataset);
- *Number of true negatives* (citations which are not duplicates which correctly remain in the dataset);
- *Number of false negatives* (citations which are duplicates which remain in the dataset but which should have been removed).

In line with other evaluations of other deduplication tools [10, 12], we calculated the *sensitivity* (true positive rate) and *specificity* (true negative rate) and the standard error of these measures [35]. To evaluate the proportion of true duplicate within the citation removed by each tool, we calculated the *precision* (positive predictive value). Given the importance of minimising false positives while maintaining a high sensitivity in deduplication tasks, we also calculated the *F1* score, the harmonic mean of precision and sensitivity. A higher F1 score (closer to 1) indicates that the tool is more sensitive and more precise. Together with sensitivity and specificity, the F1 score is one of the most commonly used performance metrics for evaluating binary (TRUE/FALSE) classification tasks [36].

$$Precision= \frac{true\ positive }{true\ positive+false\ positive}$$

$$Sensitivity = \frac{true\ positive }{true\ positive+false\ negative}$$

$$Specificity = \frac{true\ negative }{true\ negative +false\ positive}$$

$$SE (sensitivity) = \frac{sensitivity (1-sensitivity)}{true\ positive+false\ negative}$$

$$SE (specificity) = \frac{specificity (1-specificity)}{false\ positive+ true\ negative}$$

$$F1 score =2\bullet \frac{precision\bullet sensitivity}{precision+sensitivity}$$

We also recorded the time taken by each tool to deduplicate each dataset.

## Results

### True duplicates identified by any method

Across all datasets, additional duplicates were identified by automated tools which had been missed by the human reviewer(s). Furthermore, a small number of citations had been removed incorrectly by the human reviewer(s). We carefully considered all discrepancies between human reviewers and the automated tools to derive a new consensus “gold standard” annotation against which to compare all approaches.

### Diabetes dataset

The Diabetes dataset (*N* = 1845) had 1261 duplicate citations (68.3% of total; Table 4), of which 893 had been identified by human reviewers in the course of the systematic review, and a further 368 identified by at least one of the automated approaches and later confirmed by human scrutiny (c.f. Table 3). While the sensitivity of the human approach was low, the specificity was high; only three citations were removed which were not duplicates (Table 5). EndNote, the SRA-DM, and ASySD were highly effective at identifying duplicates (sensitivity = 0.966, 0.910, and 0.998 respectively), but SRA-DM had a higher rate of false positives (*n* = 70 citations incorrectly removed; 5.8% of duplicates removed incorrectly). The ASySD tool outperformed all other automated methods in terms of sensitivity (0.998), specificity (1.0), precision (1.0), and F1 score (0.999). Each automated deduplication method took less than 5 min to identify and remove duplicates in the diabetes dataset.

**Table 4:** Record classification in the Diabetes dataset by each deduplication method

Deduplication method	Duplicate citations removed	Citations remaining
TRUE duplicates (all methods + hand searching)	1261	584
Human	896	949
EndNote (automatic)	1218	627
SRA-DM	1217	628
ASySD	1259	586

**Table 5:** Performance of deduplication tools in the Diabetes dataset

	TP	TN	FN	FP	Sensitivity (SE)	Specificity (SE)	Precision	F1	Time
Human	893	581	368	3	0.708 (0.013)	0.995 (0.003)	0.997	0.828	Unknown
EndNote	1218	584	43	0	0.966 (0.005)	1 (0)	1.0	0.983	< 5 min
SRA-DM	1147	514	114	70	0.91 (0.008)	0.88 (0.013)	0.942	0.926	< 5 min
ASySD	1259	584	2	0	0.998 (0.001)	1 (0)	1.0	0.999	< 5 min

*TP* True positives, *TN* True negatives, *FN* False negatives, *FP* False positives, *SE* Standard error

### Neuroimaging dataset

The Neuroimaging dataset (*N* = 3434) had 1298 duplicate citations (37.8% of total; Table 6). In this dataset, the human reviewer was highly sensitive and identified the vast majority of duplicate citations (sensitivity = 0.985; Table 7). However, a few citations had been removed in error (*n* = 6), and a small number of duplicate citations were missed (*n* = 24). Automated deduplication by EndNote and the SRA-DM was lacking in sensitivity and each missed hundreds of duplicates (*n* = 315 and 248 respectively). The SRA-DM incorrectly removed a substantial number of citations (*n* = 42; 3.8% of duplicates removed incorrectly). The false positives rate of the ASySD (*n* = 3) and EndNote (*n* = 3) were comparable to human performance. Overall, the ASySD tool outperformed all other automated methods in terms of sensitivity (0.985), specificity (0.999), precision (0.998), and F1 score (0.991). Each method took under 5 min to identify and remove duplicates.

**Table 6:** Record classification in the Neuroimaging dataset by each deduplication method

Deduplication method	Duplicate citations removed	Citations remaining
TRUE duplicates (all methods + hand searching)	1298	2136
Human	1280	2158
EndNote (automatic)	986	2452
SRA-DM	1092	2346
ASySD	1282	2156

**Table 7:** Performance of deduplication tools in the Neuroimaging dataset

	TP	TN	FN	FP	Sensitivity (SE)	Specificity (SE)	Precision	F1	Time
Human	1274	2134	24	6	0.982 (0.004)	0.997 (0.001)	0.995	0.988	Unknown
EndNote	983	2137	315	3	0.757 (0.012)	0.999 (0.001)	0.997	0.861	< 5 min
SRA-DM	1050	2098	248	42	0.81 (0.011)	0.98 (0.003)	0.962	0.879	< 5 min
ASySD	1279	2137	19	3	0.985 (0.003)	0.999 (0.001)	0.998	0.991	< 5 min

*TP* True positives, *TN* True negatives, *FN* False negatives, *FP* False positives, *SE* Standard error

### Cardiac dataset

This cardiac dataset (*N* = 8948) contained 3530 duplicate citations (39.4% of total; Table 8). The human reviewer sensitivity was high, and they captured most duplicates (sensitivity = 0.888; Table 9). Seventeen records had been removed in error. EndNote missed a substantial portion of duplicates (sensitivity = 0.775). The SRA-DM identified many false positives (*n* = 273; 19.2% of duplicates removed incorrectly) and missed many duplicates (*n* = 2379). The ASySD tool outperformed other automated methods in terms of sensitivity (0.992) and F1 score (0.996) and was matched by EndNote in precision (0.999). Deduplication took less than 5 min using EndNote or ASySD and just under 30 min using the SRA-DM.

**Table 8:** Record classification in the Cardiac dataset by each deduplication method

Deduplication method	Duplicate citations removed	Citations remaining
TRUE duplicates (all methods + hand searching)	3530	5418
Human	3153	5795
EndNote (automatic)	2737	6211
SRA-DM	1424	7524
ASySD	3507	5441

**Table 9:** Performance of deduplication tools in the Cardiac dataset

	TP	TN	FN	FP	Sensitivity (SE)	Specificity (SE)	Precision	F1	Time
Human	3136	5401	394	17	0.888 (0.005)	0.997 (0.001)	0.994	0.939	Unknown
EndNote	2735	5416	795	2	0.775 (0.007)	1 (0)	0.999	0.873	< 5 min
SRA-DM	1151	5145	2379	273	0.326 (0.008)	0.95 (0.003)	0.808	0.465	< 30 min
ASySD	3503	5414	27	4	0.992 (0.001)	0.999 (0)	0.999	0.996	< 5 min

*TP* True positives, *TN* True negatives, *FN* False negatives, *FP* False positives, *SE* Standard error

### Depression dataset

The depression dataset (*N* = 79,880) contained 10,135 duplicate citations (12.7% of total; Table 10). The human reviewer sensitivity was high, and they correctly identified most duplicates (*n* = 9393; sensitivity = 0.927). EndNote missed many duplicate citations (sensitivity = 0.743; Table 11) but was highly specific, removing only five duplicate citations incorrectly (specificity > 0.999). The SRA-DM was highly sensitive (sensitivity = 0.934) but removed a substantial number of false positive duplicates (*n* = 1337). Overall, ASySD had a high sensitivity (0.951), specificity (0.999), precision (0.994), and a higher F1 score (0.972) than other automated tools. Deduplication using EndNote or ASySD took less than an hour, while the SRA-DM took approximately 48 h to complete the process.

**Table 10:** Record classification in the Depression dataset by each deduplication method

Deduplication method	Duplicate citations removed	Citations remaining
TRUE duplicates (all methods + hand searching)	10,135	69,745
Human	9418	70,462
EndNote (automatic)	7536	72,344
SRA-DM	10,796	69,084
ASySD	9683	70,197

**Table 11:** Performance of deduplication tools in the Depression dataset

	TP	TN	FN	FP	Sensitivity (SE)	Specificity (SE)	Precision	F1	Time
Human	9393	69,720	742	25	0.927 (0.003)	1	0.997	0.961	Unknown
EndNote	7531	69,740	2604	5	0.743 (0.004)	1	0.999	0.852	< 30 min
SRA-DM	9462	68,411	673	1334	0.934 (0.002)	0.981	0.876	0.904	~ 48 h
ASySD	9627	69,689	508	56	0.951 (0.002)	0.999	0.994	0.972	< 1 h

*TP* True positives, *TN* True negatives; *FN* False negatives, *FP* False positives, *SE* Standard error

### Systematic review of systematic reviews dataset

The SRSR dataset (*N* = 53,001) had 16,855 duplicate citations (31.8% of total; Table 12). The human reviewer sensitivity was high (sensitivity = 0.988; Table 13), capturing nearly all duplicates and outperforming other methods. EndNote lacked sensitivity (0.758) and removed the fewest citations overall. The SRA-DM identified many false positives (*n* = 1871) and lacked sensitivity (0.709). The ASySD tool outperformed other automated methods in terms of sensitivity (0.981) and precision (0.998) and was matched by EndNote on specificity (0.999), with a low false positive rate. Manual deduplication had taken one team member (ZB) approximately 9 h to complete using EndNote. Automated deduplication via ASySD and EndNote took less than 1 h, and the SRA-DM took just under 24 h.

**Table 12:** Record classification in the SRSR dataset by each deduplication method

Deduplication method	Duplicate citations removed	Citations remaining
TRUE duplicates (all methods + hand searching)	16,855	36,146
Human	16,778	36,223
EndNote (automatic)	12,830	40,171
SRA-DM	13,814	39,187
ASySD	16,564	36,437

**Table 13:** Performance of deduplication tools in the SRSR dataset

	TP	TN	FN	FP	Sensitivity (SE)	Specificity (SE)	Precision	F1	Time
Human	16,653	36,021	202	125	0.988 (0.001)	0.997 (0)	0.993	0.990	~ 9 h
EndNote	12,784	36,100	4071	46	0.758 (0.003)	0.999 (0)	0.996	0.861	< 1 h
SRA-DM	11,943	34,275	4912	1871	0.709 (0.003)	0.948 (0.001)	0.865	0.779	< 24 h
ASySD	16,529	36,111	326	35	0.981 (0.001)	0.999 (0)	0.998	0.989	< 1 h

*TP* True positives, *TN* True negatives, *FN* False negatives, *FP* False positives, *SE* Standard error

### Overall performance

**Fig. 1:**
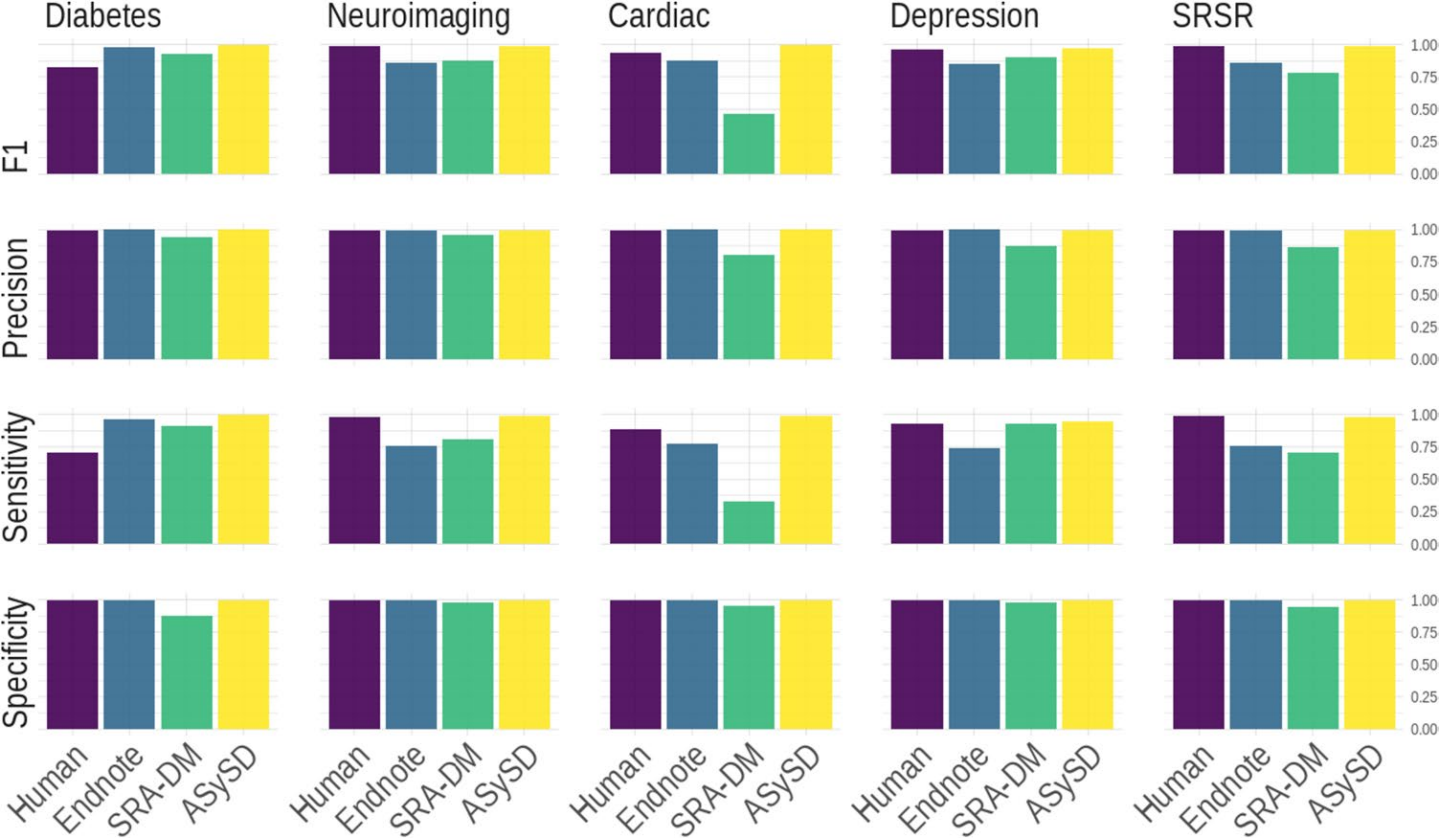
Overall performance of deduplication methods

Across all datasets, EndNote’s automated deduplication function and ASySD had consistently low false-positive rates and high specificity. ASySD correctly identified more duplicate citations than EndNote (and often more than the human reviewer). SRA-DM removed more duplicates than EndNote in some cases, but the false-positive rate of SRA-DM was high. Compared with the gold standard omnibus test (candidate duplicates identified by any approach and confirmed following human scrutiny), ASySD falsely labelled 98 citations as duplicates, and human reviewers had falsely labelled 176 citations as duplicates. This gives specificity, across all 5 datasets of 0.999 for ASySD and 0.998 for human reviewers, and sensitivity of 0.973 for ASySD and 0.948 for human reviewers (Fig. 1).

## Discussion

We developed ASySD to address the need for an effective, user friendly, low-effort, and transparent deduplication tool for biomedical systematic searches. When comparing the performance of ASySD to other tools offering similar automated features (Endnote and SRA-DM), ASySD outperformed the alternative methods, correctly removing > 95% of duplicate citations across five biomedical datasets, while removing few citations incorrectly (specificity > 0.999).

### Human error/discrepancies

We evaluated the performance of different deduplication approaches using datasets from past and existing systematic review projects that were not specifically established to test a deduplication tool. The rationale by which a reviewer removed any given citation is therefore not clear, and there are a number of possible reasons: accidental deletion, removal due to knowledge that article was not relevant, or corrupted files. The process is also likely to be influenced by differences in how reviewers determine what a duplicate is. For example, reviewers may decide to remove conference abstracts when there is a later publication with the same title and authors. Information on what would be classed as a “duplicate” was only present in one of the corresponding gold-standard search protocols/publications. For the depression review [37], publications identified in the systematic search which reported the same primary data were considered duplicates, which diverges from our definition.

### Dataset variability

We aimed to test each tool on various search datasets (i.e. differing in size and number of duplicates) to determine which tool may work best for different systematic reviews. EndNote’s lack of sensitivity was not immediately apparent on the smallest dataset (Diabetes) but was clearly shown in larger datasets. With the exception of the Diabetes dataset, the sensitivity and specificity of EndNote was fairly consistent across all datasets. The sensitivity and specificity of ASySD was also consistent, indicating that size of dataset and duplicate proportion do not seem to affect performance. SRA-DM varied in performance, with no clear explanatory pattern emerging.

### False positives

While ASySD and EndNote maintained low false positive rates, SRA-DM had a much larger false-positive rate. The SRA-DM was developed on clinical systematic review search datasets, which may differ in key matching criteria or other characteristics. Furthermore, it was previously assessed on 4 relatively small (1000 s rather than 10,000 s) datasets of fewer than 2000 citations, which may have masked the issue. However, we did not observe trends to suggest that performance was better in smaller datasets compared to larger datasets. We noticed that citations were often removed where they were recorded as having the same DOI. This can occur when a publisher assigns a single DOI to a collection of for instance conference abstracts. In such instances, inspection of the title showed that the works were clearly independent, and not duplicates. The datasets where this was the biggest problem were also the datasets with the highest proportion of duplicates (Cardiac and Diabetes datasets). With any highly sensitive automated deduplication tool, there will likely be some margin of error and a small number of citations removed incorrectly. More work is needed to understand the impact of false duplicates in different use cases and on each step of the systematic review process. For example, a recent evaluation across commonly used deduplication tools identified that a greater proportion of false positives were non-primary research articles (e.g. review articles, opinion pieces) versus primary research [10].

### Time and practicality

Stand-alone deduplication tools have been deemed as impractical [5] due to the need to import large files to an external application (i.e. not a citation manager). This will likely depend on the needs of a given review and the relative importance of sensitivity, resource requirements, and interoperability concerns. EndNote and ASySD were the fastest methods of deduplication, with all datasets taking under an hour to complete. SRA-DM was extremely slow for larger datasets. However, the interface was user-friendly and if a reviewer is not short of time, the program can run easily in the background without demanding too much processing power.

### Limitations and future directions

ASySD was developed exclusively using preclinical systematic review datasets. One dataset tested here (Neuroimaging dataset) had both clinical and preclinical studies; however, performance has not been evaluated thoroughly on systematic searches within other review areas. In the future, it would be beneficial to assess performance on additional types of systematic review datasets.

Due to the matching algorithm, the accuracy of ASySD is highly dependent on the quantity and quality of citation information. It is unclear how any of the other tools mentioned here would perform on older searches or citations without page numbers, DOIs, ISBNs, and other useful bibliographic information. In these cases, it is likely that the code may need to be adapted or that the user would have to rely more heavily on manual deduplication. To alert users about this, there is an opportunity to view the percentage of missing data across metadata fields in the latest version of the ASySD app [38] before starting deduplication process.

Furthermore, ASySD users are likely to have different criteria for determining what counts as a “duplicate”. In future versions of ASySD, we plan to build in additional user-defined options to specify whether the algorithm should consider conference abstracts, preprints, and journal articles with very similar bibliographic information to be duplicates or not. In time, with machine-readable full-text PDFs, it may also be possible to detect the same data published across multiple publications and flag these as duplicates.

While specificity was comparable to human performance (ASySD = 0.999, human = 0.998), ASySD did remove some citations incorrectly. For smaller reviews in particular, this risk may not be acceptable as each relevant paper will carry more weight than in larger reviews. In such cases, we would advise users to download all citations and manually inspect the duplicates ASySD has identified.

A key limitation of using ASySD for larger datasets (> 50,000 citations) is that the memory requirements outstrip what is possible for a shiny app hosted on shinyapps.io. We recommend that users use the R package or run the Shiny application locally in R Studio for this purpose but recognise that this may cause problems for those who are not proficient in R. We are currently exploring the implementation of parallel processing in the web application for more efficient memory management. Alternatively, we could enable methods to provide sufficient processing efficiency, such as the development of deduplication software which could be installed locally. We expect that the ASySD application will be equipped to handle large datasets in the near future.

Finally, this research was conducted between May 2020 and April 2022. The results of this evaluation may become outdated over time as tools are updated. We are continuing to develop more functionality within ASySD and finding ways to improve performance. For example, the slight discrepancy in results described here between the default settings and specifying certain labelled citations to retain has now been resolved. However, the match filters to determine a duplicate remain unchanged since this evaluation took place. The version of ASySD used for this evaluation is available on Zenodo [39]. Significant changes in future versions will be clearly documented in the NEWS section of the application and via GitHub. We also hope to submit the R package to CRAN in the coming months to enable easier access for the R evidence synthesis community.

## Conclusions

Across five preclinical systematic search datasets of varying size and duplicate proportions, the ASySD tool outperformed the SRA-DM and EndNote in detecting duplicates and had a false-positive rate comparable to human performance. For preclinical systematic reviews, automated duplicate removal using ASySD is a highly sensitive, reliable, and time-saving approach. The ASySD tool is freely available online via an R Shiny web application and the code behind the application is open source. Further research is needed to fully evaluate and disseminate the performance of various deduplication methodologies and prioritise areas for improvement.

### Supplementary Information

**Additional file 1.** Deduplication results for each dataset using the default configuration

## Data Availability

The pre-registered protocol, systematic search datasets, results, and analysis code used during the current study are available on the Open Science Framework within components in the Automated Systematic Search Deduplicator tool (ASySD) repository available at: https://osf.io/c9evs/. The version of the ASySD application used in this evaluation is available on Zenodo available at: https://zenodo.org/record/7937612#.ZGKW4nbMJPY. All underlying code for ASySD has been formalised into an R package, to ensure it is interoperable and convenient for researchers wishing to integrate ASySD into their own automated evidence synthesis workflows. The R package is available at: https://github.com/camaradesuk/ASySD and the latest R Shiny application is available at: https://camarades.shinyapps.io/ASySD/.

## Abbreviations

ASySD: Automated Systematic Search Deduplicator
SRA-DM: Systematic Review Assistant Deduplication Module
