# Supplementary material for: The Automated Systematic Search Deduplicator (ASySD): a rapid, open-source, interoperable tool to remove duplicate citations in biomedical systematic reviews
Source: BMC Biol. 2023 Sep 7;21:189. doi: 10.1186/s12915-023-01686-z (PMC10483700; doi:10.1186/s12915-023-01686-z)
Supplement: Supplementary file 1 — Additional file 1. Deduplication results for each dataset using the default configuration [file 12915_2023_1686_MOESM1_ESM.docx]

| Dataset | Duplicate citations removed | Citations remaining |
| --- | --- | --- |
| Depression | 9,696 (+13 more) | 70,184 (-13 fewer) |
| SRSR | 16,564 (+13 more) | 36,424 (-13 fewer) |
| Diabetes | 1,259 | 586 |
| Neuroimaging | 1,282 | 2,156 |
| Cardiac | 3,507 | 5,441 |
